# Maternal Blood Group and Routine Direct Antiglobulin Testing in Neonates: Is There a Role for Selective Neonatal Testing?

**DOI:** 10.3390/children8050426

**Published:** 2021-05-20

**Authors:** Hwazen A. Shash, Suzan A. Alkhater

**Affiliations:** 1 College of Medicine, Imam Abdulrahman Bin Faisal University, Dammam 31441, Saudi Arabia; saalkhater@iau.edu.sa; 2 Department of Pediatrics, King Fahad Hospital of the University, Al-Khobar 31952, Saudi Arabia

**Keywords:** hemolytic disease of the newborn, ABO group, neonatology, screening, direct antiglobulin test

## Abstract

Recommendations for the screening of hemolytic disease of the newborn (HDN) advise taking a selective approach in using the direct antiglobulin test (DAT) for mothers with blood group O or RhD-negative. This study assessed the relation of DAT results to maternal and neonatal blood groups and evaluated the risk of HDN. A retrospective analysis of all healthy newborns admitted during 2018 was performed. Of 1463 newborns, 4.4% had a positive DAT. There were 541 (37%) maternal–neonatal pairs with ABO incompatibility, most commonly born to mothers with blood group O. The cohort of neonates born to mothers with blood group O was divided into three groups: the O-A and O-B groups and the O-O group as a control. The DAT was positive in 59 (8.3%) neonates; most were in the O-B group (49.2%), whereas 13.6% were in the control group (*p* < 0.01). While the neonates in the O-B group were more likely to require phototherapy (*p* = 0.03), this finding was not related to DAT results. We found that selective testing of mothers with blood group O, mothers with blood group O or RhD-negative, neonates with blood group B, and neonates with blood group B born to mothers with blood group O or RhD-negative was ineffective in detecting phototherapy requirements. Our results indicate no difference regarding the need for phototherapy in neonates born to mothers with different blood types regardless of the DAT results.

## 1. Introduction

Hemolytic disease of the newborn (HDN) is a condition that occurs due to IgG antibodies that cross the placenta as a result of maternal sensitization to fetal red blood cell (RBC) antigens inherited from the father, leading to hemolysis and subsequent hyperbilirubinemia [1,2]. It can occur due to Rh antigens, most commonly the D antigen, and ABO antigens. RhD-HDN has decreased since the introduction of antenatal and postpartum Rh immunoglobulin (anti-D IgG or RhoGAM); therefore, ABO incompatibility is now the most common cause of HDN [2].

ABO-HDN is described as having a low incidence with a more benign presentation and outcome. The main clinical presentation is jaundice and, rarely, anemia [3]. Early diagnosis and treatment of neonatal hyperbilirubinemia are essential in the prevention of kernicterus [4]. ABO-HDN is almost exclusive to mothers with blood group O, as the naturally occurring antibodies are of the IgG subtype. This contrasts with individuals with blood group A or B, where the antibodies are predominantly IgM and do not cross the placenta [1]. The frequency of ABO incompatibility between a mother and neonate and the severity of HDN vary between ethnicities [3,5]. The literature differs on the type of ABO incompatibility, and whether maternal group O/neonate group A (O-A) or maternal group O/neonate group B (O-B) is associated with a more severe presentation [6,7,8,9].

The direct antiglobulin test (DAT) is a screening test used to determine if antibodies are attached to a patient’s RBCs [10]. There are different approaches to the use of the DAT to screen neonates at increased risk of HDN. The DAT can detect antigen–antibody binding; however, it may not predict the affected neonate’s clinical course [3]. Routine testing for all newborns was a common practice [6]. However, current recommendations advocate for more selective testing—to be considered only in mothers with blood group O or RhD-negative—while others encourage testing according to the clinical evaluation of newborns [11,12].

As the literature describes various methods for using the DAT to asses for HDN, our aim was to evaluate the need for routine DAT screening in healthy full-term newborns in our predominantly Arab population by correlating all maternal–neonatal blood types, bilirubin levels, and DAT findings with the need for phototherapy. We aimed to evaluate the blood groups in mothers and neonates and determine the combinations at increased risk of HDN. There is currently no unified approach to screening for HDN in Saudi Arabia. The centers perform either universal screening, selective testing, or testing according to the clinical presentation. The current protocol in our hospital is to conduct universal DAT screening for all newborns. We intended to evaluate the possibility of adopting new local screening guidelines. We determined the incidence of DAT positivity over one year and evaluated the associations between maternal ABO/Rh status, neonatal risk factors, DAT results, and the need for phototherapy within the first 48 h before discharge.

## 2. Materials and Methods

A retrospective chart review was performed on all newborns admitted to the nursery at King Fahad Hospital of the University, Al-Khobar, Saudi Arabia, a tertiary care university hospital with approximately 1500 births per year. Ethical approval for the study was obtained from the university institutional review board (IRB-UGS-2019-01-340, December 2019). We collected data on maternal–neonatal pairs between 1 January and 31 December 2018, from electronic medical records and blood bank records. The ABO/Rh status evaluation, DAT results and strength, and antibody identification were performed by the Dia-Med gel testing method (Bio-Rad Laboratories, Cressier, Switzerland) [13].

The data extracted from the mothers’ files included age, ethnicity, parity, mode of delivery, ABO/Rh group, whether anti-D was given to RhD-negative mothers, and maternal antibody screening and identification. The data extracted for the newborns included sex, gestational age in weeks, birthweight in kilograms, ABO/Rh group, and DAT results from cord blood. If the cord sample was clotted, the tests were obtained from the serum. We further determined the antibody eluate detected and DAT positivity strength in newborns with a positive DAT result. The strength is a graded scale from 0 to 4+ depending on the degree of agglutination, where 0 indicates no agglutination and 4+ indicates solid agglutination [14]. The standard of practice in our institution is to discharge full-term vaginal deliveries at 24 to 48 h of age, neonates born to mothers after cesarean section at 72 to 96 h, and neonates with lab investigations performed per hospital protocol (for small for gestational age, DAT positivity, infant of diabetic mother) at variable times according to investigation results.

There were three groups of newborns for which the serum bilirubin level was obtained. First, all the patients with a DAT-positive result had their bilirubin level determined, per the hospital protocol, at 3 h and 24 h of age. Second, the hospital protocol requires bilirubin levels to be determined at 24 h of age in all infants of diabetic mothers and small for gestational age infants, regardless of their clinical condition. Finally, the bilirubin level was also obtained for any newborn during the nursery stay if significant clinical jaundice was noted on the nurse’s or physician’s clinical examination. Determination of bilirubin levels for all groups was repeated as clinically indicated. The bilirubin levels were plotted on an hour-specific bilirubin nomogram based on the National Institute for Health and Clinical Excellence (NICE) guidelines to evaluate the need for and type of phototherapy [15].

We studied the ABO blood groups of the maternal–neonatal pairs and determined whether they were both of the same blood group (blood group-compatible) or had different blood groups (blood group-incompatible). We also evaluated Rh compatibility in the same manner. We compared all the blood groups to each other and neonatal blood group A or B to maternal blood group O.

Statistical analysis was performed using SPSS version 24. Univariate comparisons of variables were performed with the independent *t*-test (uniformly distributed data) and the Mann–Whitney U test for continuous variables. The categorical variables were compared by the chi-square test (with the Yates continuity correction) or the Fisher exact test. The results are presented as the mean (standard deviation) or median (interquartile range) depending on the normality of the data distribution. Data normality was tested using the Shapiro–Wilk test. Multiple logistic regression with the therapeutic use of phototherapy as the dependent variable was conducted as part of the analysis in another report [16].

## 3. Results

There were 65 (4.4%) neonates with DAT-positive results in the entire cohort of 1463 maternal–neonate pairs. There was no difference in maternal age, gestational age, or delivery mode when DAT-positive and DAT-negative neonates were compared. Although there was no difference in neonatal gender, DAT-positive babies had a larger birthweight (*p* = 0.002) (Table 1). Saudi mothers comprised 82.1% of the cohort, with the remaining patients being of multiple other ethnicities, including Asian, African, and Indian.

Phototherapy was required in 36 neonates (2.46%). The results of the phototherapy analysis are described in another study [16]. The most common indication for phototherapy was hyperbilirubinemia detected by clinical jaundice (61.1%), followed by DAT-positive results (33.3%). None of the newborns required immunoglobulin infusion or exchange transfusion.

### 3.1. DAT and Maternal ABO Groups

The most common maternal blood group was blood group O (49.9%), followed by group A (27.1%) (Table 2). There were 541 (37%) maternal–neonatal pairs with ABO incompatibility. These neonates were most commonly born to mothers with blood group O (36.4%), followed by mothers with blood group A (32.3%) (Table 2). When comparing DAT positivity in blood group-compatible and incompatible neonates, the frequencies were 1.1% (10/922) and 10.2% (55/541), respectively (*p* < 0.001). Of the 10 neonates who were ABO-compatible with a positive DAT, 9 had maternal antibodies detected in the eluate. There were two neonates with anti-D, three with anti-E, one with anti-c and three with a nonspecific antibody. A nonspecific antibody was detected in the only patient in this cohort who required phototherapy.

**Figure 1:**
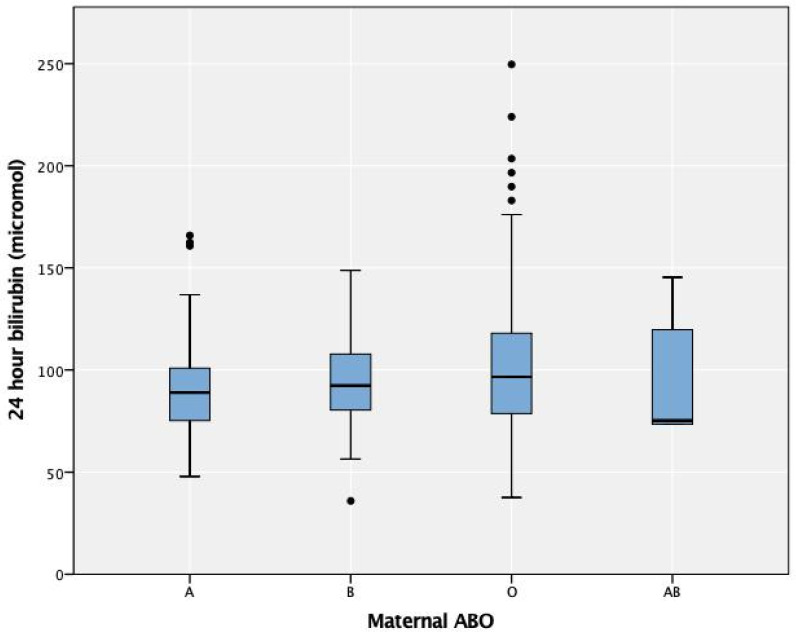
Twenty-four-hour serum bilirubin by maternal ABO blood groups. The circles represent the outliers of the bilirubin levels in each type of blood group.

The most common maternal blood group with neonatal DAT-positive results was blood group O (90.8%), with none in mothers with blood group A (Table 2). There was no difference in the 24-h serum bilirubin level in the DAT-positive and DAT-negative groups in regard to different maternal blood groups (*p* = 0.412) (Figure 1) or maternal–neonatal ABO incompatibility (*p* = 0.329). There was no difference in the phototherapy requirement between all the maternal blood groups, regardless of the DAT results (*p* = 0.54).

### 3.2. DAT and Maternal Group O

**Figure 2:**
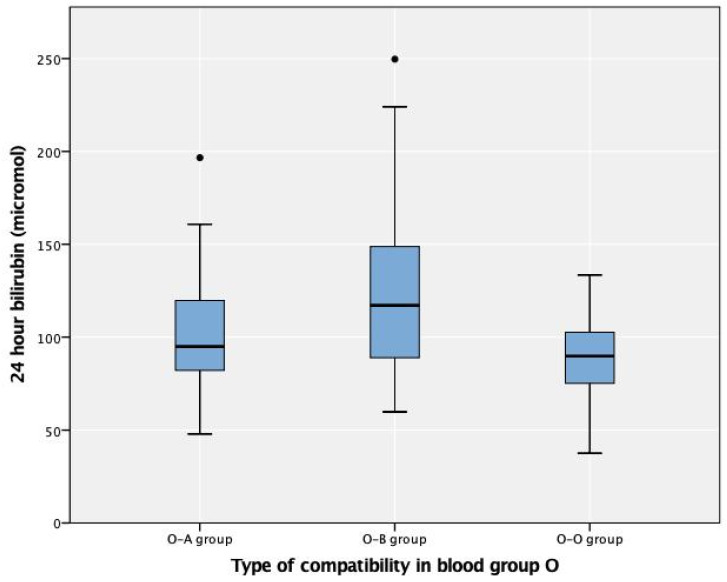
Twenty-four-hour bilirubin in neonates born to mothers with blood group O. The circles represent the outliers of the bilirubin levels in each type of blood group incompatibility.

Of the 722 mothers with blood group O, 197 (27.3%) had ABO incompatibility (Table 3). We divided the cohort into three groups: 15.4% in the O-A group, 11.9% in the O-B group, and the remaining in the O-O group, which represented a control. There were 59 (8.3%) neonates with a positive DAT, most commonly in the O-B group (49.2%), with 13.6% in the control group (*p* < 0.01). There were 106 neonates with 24-h serum bilirubin levels reported, with the highest value of 123 µmol/L (7.2 mg/dL) in the O-B group and the lowest in the control group (*p* = 0.001) (Figure 2). The need for phototherapy was assessed in 143 neonates: 41.3% (59/143) due to a positive DAT, 23.1% (33/143) due to clinical jaundice, and 35.7% (51/143) per the hospital protocol. Phototherapy was required in 22 of the 143 (15.4%) neonates, of which half had a positive DAT (*p* = 0.5) and 72% (16/22) had ABO incompatibility (*p* = 0.05). While neonates in group O-B were more likely to require phototherapy than those in the other groups (*p* = 0.03, chi-square test), this finding was not related to the DAT results in the binary logistic regression. The most common antibody detected in newborn sera was anti-B (50%), followed by anti-A (36.2%).

### 3.3. DAT and Maternal RhD Groups

There were 92 (6.3%) mothers who were RhD-negative, with 66.3% reportedly having received anti-D at an unknown timing (Table 2), and 58 (47%) had RhD incompatibility. There was no difference in the DAT results in relation to maternal RhD status (*p* = 0.239). There were nine (9.8%) DAT-positive neonates, of which five (55.6%) mothers had received anti-D IgG. None of these newborns required phototherapy. Only one RhD-positive and DAT-negative neonate (1%) required phototherapy at 48 h of life when noted to be clinically jaundiced.

### 3.4. High-Risk Groups

In our study, of the 768 neonates delivered to O-positive or RhD-negative mothers, which theoretically have the highest risk of developing HDN, 8.2% (63/768) had a positive DAT and 2.9% (23/768) required phototherapy. Half of the neonates who required phototherapy had blood group B (52.2%). There was no difference in the phototherapy requirement in relation to the DAT results in this subgroup (*p* = 0.46).

Thirty-six neonates required phototherapy, and the group most frequently requiring phototherapy in our study were neonates of blood group B (18/36, 50%). We hypothesized that performing a routine DAT on different selective groups might detect ≥80% of the 36 newborns that required phototherapy (Supplementary Tables S1 and S2). We studied selective testing of mothers with blood group O, mothers with blood group O or RhD-negative, neonates with blood group B, and neonates with blood group B born to mothers with blood group O or RhD-negative. We found that selective testing was not helpful in detecting the phototherapy requirement in any group, regardless of the DAT results.

## 4. Discussion

Our results indicate no difference regarding the need for phototherapy in neonates born to mothers of different blood types regardless of the DAT results. Positive DAT results were most common in maternal blood group O, and there was no difference in the requirement of phototherapy in this subgroup regardless of the DAT results.

ABO incompatibility between mother and neonate occurs in 15–20% of all pregnancies, occurring in one in every five pregnancies [17,18]. However, this occurred in 37% of the maternal–neonate pairs in our cohort, approximately one in every three (2.7) pairs. This frequency is greater than that in the Caucasian population and similar to the results reported in Venezuela [5]. HDN occurs at different frequencies in relation to ethnicity, with reports of it being more common in Blacks and South Americans [3,19,20]. Jawad et al. concluded that HDN in Arabs was approximately as common as that in Blacks and less common than that in Caucasians [21]. Our hospital is a governmental hospital that mainly treats Saudi citizens; therefore, they were more commonly seen. There were multiple other ethnicities represented in this study, but there were too few cases of each ethnicity to study the effect on DAT results and subsequent phototherapy.

The most common blood group in Saudi Arabia is blood group O, followed by blood group A, which was evident in our results, with the number of neonates in the O-A group being greater than that in the O-B group [22]. However, the DAT results and phototherapy requirements were higher in the O-B group. Murray et al. described that hemolysis due to anti-A is more common than that due to anti-B [6]. The number of symptomatic cases in several studies was higher in group O-A [6]. This was contrary to the results of other researchers who found that symptomatic cases were more common in the O-B group [8,9]. None of these papers showed differences in the need for phototherapy between the groups [7,8]. Multiple other studies showed no significant differences in hemolysis or the need for phototherapy between groups O-A and O-B [23,24]. These studies are from different countries representing varying ethnicities, which may suggest the different nature of the antibodies and strength of binding.

RhD incompatibility was previously the most common cause of HDN. As most of the mothers’ follow-up was mainly in primary health care centers, the status and timing of receiving anti-D IgG were not available in most cases. Anti-D IgG administration to the mother, especially if given within 6 weeks before delivery, is associated with a positive DAT in the newborn [25,26]. The placental passage of immunoglobulin may not cause hematologic changes and hemolysis, even if near delivery [26]. The anti-D IgG titer level in the fetus may not be elevated to the level of causing hemolysis, even if the antibody is detected in neonatal sera. This lack of hemolysis was also shown previously in a study of RhD-positive adults who received anti-D IgG [27]. This may explain the five mothers with anti-D detected during the antibody screen. All neonates born to these mothers had a positive DAT, and none required phototherapy.

The American Academy of Pediatrics (AAP) recommends two clinical options used individually or in combination for risk assessment: universal screening with transcutaneous bilirubin or serum bilirubin levels and/or assessment of clinical risk factors [11]. They describe ABO incompatibility and a positive DAT in neonates as a risk factor; however, they consider testing optional provided there are predischarge risk assessments and follow-ups. The AAP recommendation is to perform a predischarge bilirubin screen by transcutaneous bilirubin or serum bilirubin in neonates, particularly in those discharged at less than 72 h of age. As we did not determine predischarge bilirubin levels and our neonates were usually discharged at approximately 24–36 h of age, we performed a logistic regression on those requiring phototherapy in the 174 patients with available 24-h bilirubin results. A higher bilirubin level was an indicator of the need for phototherapy (*p* < 0.01). We hypothesized that different groups are considered at high risk and may benefit from selective testing and found that the DAT result was not useful in any of the groups in detecting an increased risk of developing HDN.

The NICE guidelines advise against the routine use of the DAT and focus mostly on clinical risk factors [15]. The NICE guidelines focus on clinical examination of neonatal jaundice for the need for bilirubin measurement. Studies are contradictory regarding physicians’ ability to detect clinically significant jaundice accurately [28,29]. Risken et al. studied the reliability of visual assessment of bilirubin as a screening tool and concluded that visual assessment is unreliable as a screening tool to detect clinically significant jaundice with relatively high false negative rates [30]. Surprisingly, in their study, there was a tendency to overestimate jaundice in dark-skinned children and to underestimate it in fair-skinned children [30]. Several methods attempted to increase the sensitivity and specificity of visual assessment of jaundice in order to be utilized in low-income countries, such as the two-color Bilistrip and the six-color Bili-Ruler [31,32,33]. Currently, in the era of smartphones, different applications for detecting jaundice are being developed such as the BiliCam [34]. Our population is heterogeneous, where children have variable skin colors, and health care providers have different training levels. This may reduce the accuracy of diagnosing clinical jaundice. The use of the objective measurement of bilirubin by a transcutaneous bilirubinometer, when available, is considered the current standard of care for screening of neonatal jaundice, as recommended by the AAP [11]. We could not compare the timing of developing clinical jaundice in DAT-positive and DAT-negative newborns in our study, as the bilirubin level was taken routinely in DAT-positive newborns, and they were kept under close observation, leading to bias in the timing of noticing jaundice. Our population is heterogeneous, with children with variable skin colors and health care providers with different training levels, which may lead to difficulty in evaluating clinical jaundice accurately. We also have poor follow-up compliance by parents. A prospective study would be valuable to evaluate whether predischarge bilirubin or clinical assessment of jaundice is more suitable in our population.

Our study covered an entire year, and we included all maternal–neonatal pairs, but the sample size was relatively small because it was from a single center. However, we had appropriate data regarding blood grouping and phototherapy requirements. Despite being in a region with increased hemoglobinopathies, hemoglobin H disease and G6PD deficiency are not tested as part of neonatal screening or routinely evaluated in neonates starting on phototherapy. In addition, documentation of the timing of clinical jaundice in patients, particularly DAT-positive neonates, is not optimal. There may be selection bias in neonates with clinical jaundice, likely due to differences in nurses’ or clinicians’ experience. This is indicated by the age and range of the first bilirubin level detected in relation to the degree of clinical jaundice (9–72 h and 77–302 µmol/L, respectively). The NICE guidelines for phototherapy are not strictly followed, and some patients are started on phototherapy at levels below the phototherapy cutoff. We also do not have complete records of the post-discharge follow-ups of babies who may have required phototherapy later, since some neonates may have presented to other hospitals. All the 27 babies who presented to the emergency department for evaluation of clinical jaundice after discharge were DAT-negative. The group O-O constituted 48% of the babies, and five (18.5%) required phototherapy.

## 5. Conclusions

Our study indicates that the DAT is overused in our center and universal screening is not useful in our population. Given the unique characteristics of our population, a combination approach based on both the AAP and NICE guidelines is suggested. Therefore, we recommend re-evaluating the local hospital protocols. Considering our specific regional factors, a further prospective multicenter study is needed in Saudi Arabia to unify the approach for screening HDN.

## Figures and Tables

**Table 1:** Demographic and clinical characteristics of study population by DAT results (n = 1463). *p*-Value calculated by chi-square for categorical data, and Mann–Whitney U test for continuous variables.

	Negative (n = 1398)	Positive (n = 65)	*p*-Value	Overall (n = 1463)
Maternal Age (y)				
Median (25th, 75th percentile)	30.0 (26, 35)	31.0 (27, 35)	0.556	30.0 (26.0, 35.0)
Missing	1 (0.1%)	0		1 (0.1%)
Parity				
Median (25th, 75th percentile)	2.00 (2.00, 4.00)	3.00 (1.00, 4.0)	0.549	2.00 (1.00, 12.0)
Missing	54 (3.9%)	1 (1.5%)		55 (3.8%)
Gestational Age (Categorical)				
≤36–38 + 6	520 (37.2%)	23 (35.4%)	0.444	543 (37.1%)
39–40 + 6	654 (46.8%)	26 (40.0%)		680 (46.5%)
>41	119 (8.5%)	8 (12.3%)		127 (8.7%)
Missing				113
Neonate Gender				
Male	713 (51.0%)	37 (56.9%)	0.420	750 (51.3%)
Female	685 (49.0%)	28 (43.1%)		713 (48.7%)
Birthweight (kg)				
Median	3.00 (2.7, 3.3)	3.2 (2.9, 3.6)	0.002	3.03 (0.470)
Missing	157 (11.2%)	10 (15.4%)		167 (11.4%)
Ethnicity				
Saudi	1155 (82.6%)	46 (70.8%)	0.023	1201 (82.1%)
Non-Saudi	243 (17.4%)	19 (29.2%)		262 (17.9%)

**Table 2:** Maternal blood groups and maternal–neonate compatibility by DAT. *p*-Value calculated by chi-square or Fisher exact test.

	DAT-Negative (n = 1398)	DAT-Positive (n = 65)	*p*-Value	Overall (n = 1463)
Maternal ABO				
Blood group O	663 (47.4%)	59 (90.8%)	<0.001	722 (49.4%)
Blood group A	396 (28.3%)	0 (0%)		396 (27.1%)
Blood group B	268 (19.2%)	5 (7.7%)		273 (18.7%)
Blood group AB	71 (5.1%)	1 (1.5%)		72 (4.9%)
Maternal RhD				
Positive	1315 (94.1%)	56 (86.2%)	0.021	1371 (93.7%)
Negative	83 (5.9%)	9 (13.8%)		92 (6.3%)
Received Anti-D (n = 92)				
Yes	56 (67.5%)	5 (55.6%)	0.013	61 (66.3%)
No	18 (21.7%)	0 (0%)		18 (19.6%)
Data not available	9 (10.8%)	4 (44.4%)		13 (14.1%)
Maternal/Neonate ABO Compatibility				
ABO-compatible	912 (65.2%)	10 (15.4%)	<0.001	922 (63.0%)
ABO-incompatible	486 (34.8%)	55 (84.6%)		541 (37.0%)
Type of Maternal/Neonate Incompatibility (n = 541)				
Maternal A/Baby O	125 (25.7%)	0 (0%)	<0.001	125 (23.1%)
Maternal A/Baby B	26 (5.3%)	0 (0%)		26 (4.8%)
Maternal A/Baby AB	24 (4.9%)	0 (0%)		24 (4.4%)
Maternal B/Baby O	66 (13.6%)	2 (3.6%)		68 (12.6%)
Maternal B/Baby A	24 (4.9%)	0 (0%)		24 (4.4%)
Maternal B/Baby AB	20 (4.1%)	1 (1.8%)		21 (3.9%)
Maternal O/Baby A	89 (18.3%)	22 (40%)		111 (20.5%)
Maternal O/Baby B	57 (11.7%)	29 (52.7%)		86 (15.9%)
Maternal AB/Baby A	25 (5.1%)	0 (0%)		25 (4.6%)
Maternal AB/Baby B	30 (6.2%)	1 (1.8%)		31 (5.7%)
Maternal/Neonatal RhD Compatibility				
RhD-compatible	1284 (91.8%)	57 (87.7%)	0.340	1341 (91.7%)
RhD-incompatible	114 (8.2%)	8 (12.3%)		122 (8.3%)

**Table 3:** DAT results in maternal group O. *p*-Value calculated by chi-square for categorical data, and Mann–Whitney U test for continuous variables.

	DAT-Negative (n = 663)	DAT-Positive (n = 59)	*p*-Value	Overall (n = 722)
Maternal/Neonatal Grouping				
Group O-A	89 (13.4%)	22 (37.3%)	<0.001	111 (15.4%)
Group O-B	57 (8.6%)	29 (49.2%)		86 (11.9%)
Group O-O	517 (78%)	8 (13.6%)		525 (72.7%)
24-h Bilirubin Level				
Median [25th, 75th percentile]	92.34 (77, 107.22)	106 (82.1, 134.2)	0.014	
Peak Bilirubin Level				
Median [25th, 75th percentile]	111.2 (88.9, 175.3)	126.5 (89.4, 163.3)	0.402	
Age at Peak Bilirubin				
Median [25th, 75th percentile]	25.00 (24.00, 52.00)	36.00 (24, 50)	0.569	
Phototherapy (n = 143)				
Yes	11 (13.1%)	11 (18.6%)	0.503	22 (15.4%)
No	73 (86.9%)	48 (81.4%)		212 (84.6%)

## Data Availability

The data presented in this study are available on request from the corresponding author.

## Supplementary Materials

The following are available online at https://www.mdpi.com/article/10.3390/children8050426/s1, Table S1: Selective high-risk group testing, Table S2: Percent of neonates requiring phototherapy detected in high-risk groups.

## References

[1] Kumar A., Patel M.K., Chavda B., Ranjan A., Ahmad F. (2013). Hemolytic Disease of the Newborn: A study of 50 cases. Int. J. Sci. Study.

[2] Akanmu A.S., Oyedeji O.A., Adeyemo T.A., Ogbenna A.A. (2015). Estimating the Risk of ABO Hemolytic Disease of the Newborn in Lagos. J. Blood Transfus..

[3] Klein H.G., Anstee D.J. (2014). Mollison’s Blood Transfusion in Clinical Medicine.

[4] Hendrickson J.E., Delaney M. (2016). Hemolytic disease of the fetus and newborn: Modern practice and future investigations. Transfus. Med. Rev..

[5] Cariani L., Romano E.L., Martínez N., Montaño R., Suarez G., Ruiz I., Soyano A. (1995). ABO-haemolytic disease of the newborn (ABO-HDN): Factors influencing its severity and incidence in Venezuela. J. Trop. Pediatr..

[6] Murray N.A., Roberts I.A. (2007). Haemolytic disease of the newborn. Arch. Dis. Child. Fetal Neonatal. Ed..

[7] Dufour D.R., Monoghan W.P. (1980). ABO hemolytic disease of the newborn. A retrospective analysis of 254 cases. Am. J. Clin. Pathol..

[8] Kaplan M., Hammerman C., Vreman H.J., Wong R.J., Stevenson D.K. (2010). Hemolysis and hyperbilirubinemia in antiglobulin positive, direct ABO blood group heterospecific neonates. J. Pediatr..

[9] Bel Hadj I., Boukhris R., Khalsi F., Namouchi M., Bougmiza I., Tinsa F., Hamouda S., Boussetta K. (2019). ABO hemolytic disease of newborn: Does newborn’s blood group a risk factor?. Tunis. Med..

[10] Keir A., Agpalo M., Lieberman L., Callum J. (2015). How to use: The direct antiglobulin test in newborns. Arch. Dis. Child. Educ. Pract..

[11] Subcommittee on Hyperbilirubinemia (2004). Management of hyperbilirubinemia in the newborn infant 35 or more weeks of gestation. Pediatrics.

[12] Keren R., Bhutani V.K., Luan X., Nihtianova S., Cnaan A., Schwartz J.S. (2005). Identifying newborns at risk of significant hyperbilirubinaemia: A comparison of two recommended approaches. Arch. Dis. Child..

[13] BioRad-ABO/Rh for Newborns. http://www.diamed.com/product_detail.aspx?id=99&navvis=yes.

[14] Parker V., Tormey C.A. (2017). The direct antiglobulin test: Indications, interpretation, and pitfalls. Arch. Pathol. Lab. Med..

[15] National Institute for Health and Care Excellence (2010). Jaundice in Newborn Babies under 28 Days.

[16] AlKhater S.A., Albalwi R.A., Alomar S.A., Alsultan A.A., Almuhaidib H.R., Almousa R.A., Alanezi S.M., Alghamdi R.K., Shash H.A. (2021). Value of the Direct Antiglobulin Test in Predicting the Need for Phototherapy in Newborns. J. Blood Med..

[17] Roberts I.A. (2008). The changing face of haemolytic disease of the newborn. Early Hum. Dev..

[18] Dinesh D. (2005). Review of positive direct antiglobulin tests found on cord blood sampling. J. Paediatr. Child Health.

[19] Bucher K.A., Patterson A.M., Elston R.C., Jones C.A., Kirkman H.N. (1976). Racial difference in incidence of ABO hemolytic disease. Am. J. Public Health.

[20] Huntley C.C., Lyerly A.D., Littlejohn M.P., Rodriguez-Trias H., Bowers G.W. (1976). ABO hemolytic disease in Puerto Rico and North Carolina. Pediatrics.

[21] Al Jawad S., Keenan P., Kholeif S. (1986). Incidence of ABO haemolytic disease in a mixed Arab population. Saudi Med. J..

[22] Bashwari L., Al-Mulhim A.A., Ahmad M.S., Ahmed M.A. (2001). Frequency of ABO blood groups in the Eastern region of Saudi Arabia. Saudi Med. J..

[23] Akgül S., Korkmaz A., Yiğit S., Yurdakök M. (2013). Neonatal hyperbilirubinemia due to ABO incompatibility: Does blood group matter?. Turk. J. Pediatr..

[24] Bhat Y.R., Kumar C.G. (2012). Morbidity of ABO haemolytic disease in the newborn. Paediatr. Int. Child Health.

[25] Dillon A., Chaudhari T., Crispin P., Shadbolt B., Kent A. (2011). Has anti-D prophylaxis increased the rate of positive direct antiglobulin test results and can the direct antiglobulin test predict need for phototherapy in Rh/ABO incompatibility?. J. Paediatr. Child Health.

[26] Maayan-Metzger A., Leibovitch L., Schushan-Eisen I., Morag I., Strauss T. (2014). Maternal anti-D prophylaxis during pregnancy and risk of hemolysis among preterm infants. J. Perinatol..

[27] Chown B., Bowman J.M., Pollock J., Lowen B., Pettett A. (1970). The effect of anti-D IgG on D-positive recipients. Can. Med. Assoc. J..

[28] Moyer V.A., Ahn C., Sneed S. (2000). Accuracy of clinical judgment in neonatal jaundice. Arch. Pediatr. Adolesc. Med..

[29] Riskin A., Kugelman A., Kuglman A., Abend-Weinger M., Green M., Hemo M., Bader D. (2003). In the eye of the beholder: How accurate is clinical estimation of jaundice in newborns?. Acta Paediatr..

[30] Riskin A., Tamir A., Kugelman A., Hemo M., Bader D. (2008). Is visual assessment of jaundice reliable as a screening tool to detect significant neonatal hyperbilirubinemia?. J. Pediatr..

[31] Hulzebos C.V., Vitek L., Zabetta C.D.C., Dvořák A., Schenk P., van der Hagen E.A., Cobbaert C., Tiribelli C. (2021). Screening methods for neonatal hyperbilirubinemia: Benefits, limitations, requirements, and novel developments. Pediatr. Res..

[32] Olusanya B.O., Slusher T.M., Imosemi D.O., Emokpae A.A. (2017). Maternal detection of neonatal jaundice during birth hospitalization using a novel two-color icterometer. PLoS ONE.

[33] Lee A.C., Folger L.V., Rahman M., Ahmed S., Bably N.N., Schaeffer L., Whelan R., Panchal P., Rahman S., Roy A.D. (2019). A novel Icterometer for hyperbilirubinemia screening in low-resource settings. Pediatrics.

[34] Taylor J.A., Stout J.W., de Greef L., Goel M., Patel S., Chung E.K., Koduri A., McMahon S., Dickerson J., Simpson E.A. (2017). Use of a smartphone app to assess neonatal jaundice. Pediatrics.

