# Supplementary material for: Maternal Blood Group and Routine Direct Antiglobulin Testing in Neonates: Is There a Role for Selective Neonatal Testing?
Source: Children (Basel). 2021 May 20;8(5):426. doi: 10.3390/children8050426 (PMC8161132; doi:10.3390/children8050426)
Supplement: Supplementary file 1 [file children-08-00426-s001.zip › children-1201352-supplementary.pdf]

Supplementary

Table S1. Selective High Risk Group Testing.

	DAT negative	DAT positive	P value	Total
**Group 1. Screening in mothers blood group O or Rh negative (n=768)***				
Phototherapy (n=154)#				
Yes	12 (13.2%)	11 (17.5%)	0.497	23 (14.9%)
No	79 (86.8%)	52 (82.5%)		131 (85.1%)
**Group 2. Screening in mothers with blood group O (n=722)***				
Phototherapy (n=143)#				
Yes	11 (13.1%)	11 (18.6%)	0.5	22 (15.4%)
No	73 (86.9%)	48 (81.4%)		212 (84.6%)
**Group 3. Screening in neonates with blood group B (n=303)***				
Phototherapy (n=81)#				
Yes	9 (18.4%)	9 (28.1%)	0.413	18 (22.2%)
No	40 (81.6%)	23 (71.9%)		63 (77.8%)
**Group 4. Screening in neonates with blood group B born to mothers with blood group O or Rh negative (n=96)***				
Phototherapy (n=47)#				
Yes	4 (23.5%)	8 (26.7%)	0.8	12 (25.5%)
No	13 (76.5%)	22 (73.3%)		35 (74.5%)

* Number of maternal-neonatal pairs in group; # Number of neonates assessed for phototherapy.

Table S2. Percent of neonates requiring phototherapy detected in high risk groups.

Group tested	Number of patients requiring phototherapy detected	Percent decrease in testing	Positive Predictive Value [95% CI]
All cohort	36 (100%)		
Group 1	23 (63.9%)	47.5%	17.5% [11.6 – 25.4%]
Group 2	22 (61.1%)	50.7%	18.6% [12.5 – 26.8%]
Group 3	18 (50%)	79.3%	28.1% [18.2 – 40.8%]
Group 4	12 (33.3%)	93.4%	26.7% [18.5 – 36.9%]
